# Emerging Roles of Alternative Pre-mRNA Splicing Regulation in Neuronal Development and Function

**DOI:** 10.3389/fnins.2012.00122

**Published:** 2012-08-21

**Authors:** Adam D. Norris, John A. Calarco

**Affiliations:** ^1^FAS Center for Systems Biology, Harvard UniversityCambridge, MA, USA

**Keywords:** alternative splicing, genomics, nervous system, RNA processing, gene regulation

## Abstract

Alternative pre-mRNA splicing has the potential to greatly diversify the repertoire of transcripts in multicellular organisms. Increasing evidence suggests that this expansive layer of gene regulation plays a particularly important role in the development and function of the nervous system, one of the most complex organ systems found in nature. In this review, we highlight recent studies that continue to emphasize the influence and contribution of alternative splicing regulation to various aspects of neuronal development in addition to its role in the mature nervous system.

## Introduction

The nervous system is a uniquely complex structure, composed of diverse classes of neuronal cells found in close proximity to one another. These many classes of neurons must form precise synaptic connections with other neurons that can often be separated by large distances, establishing the circuitry governing the ability to sense, interpret, and appropriately respond to stimuli. It is of great interest to understand how the developmental steps of neurogenesis, migration, pathfinding, synapse formation, and maintenance are controlled with such precision. Defects in any of these processes in humans lead to numerous cognitive and motor disabilities (Mitchell, 2011). It is also of importance to better grasp the mechanisms governing the modulation of synaptic strength and plasticity in the mature nervous system, which plays a key role in sensory adaptation, learning and memory, and other behaviors.

Diverse spatio-temporal gene regulatory mechanisms have proven vital for the control of patterning in the nervous system, including regulation of mRNA synthesis by transcription factors (West and Greenberg, 2011), the dynamic alteration of chromatin states through modifying enzymes (Ooi and Wood, 2008; Yoo et al., 2009), turnover or translational repression by microRNAs (Meza-Sosa et al., 2012), RNA decay pathways, and regulation by post-translational modifications (Fukushima et al., 2009). Alternative splicing, the process in which multiple mRNA isoforms can be generated through differential splice site selection in precursor transcripts, is an additional important mechanism of gene regulation, contributing to transcriptomic and proteomic diversity in metazoans (Nilsen and Graveley, 2010). Greater than 95% of human genes undergo alternative splicing (Pan et al., 2008; Wang et al., 2008), and disruption of splicing contributes to a number of genetic diseases (Chakarova et al., 2002; Briese et al., 2005; Winkler et al., 2005; Mordes et al., 2006; Wang and Cooper, 2007). The nervous system exhibits particularly high levels of alternative splicing (Yeo et al., 2004; Grosso et al., 2008). Indeed, a recent large scale study of human tissues found that the cerebellum exhibited the highest degree of alternative splicing among 11 tested tissue samples, containing 50% more differentially expressed alternative exons than the next highest tissue (the testes; de la Grange et al., 2010). These results suggest that regulated splicing can serve as a potential mechanism for generating the high levels of molecular and cellular diversity observed in the nervous system (Lipscombe, 2005; Li et al., 2007).

The fidelity and efficiency of splicing depends on the action of five small nuclear RNAs (snRNAs) functioning as components of ribonucleoprotein particles called snRNPs, in conjunction with up to hundreds of additional auxiliary proteins (Wahl et al., 2009). This elaborate and highly dynamic complex known as the spliceosome regulates splicing with single nucleotide precision (Will and Luhrmann, 2011). Decades of research have begun to elucidate the “splicing code,” the complete set of *cis*-acting RNA features (for example, sequence motifs, exon and intron length, secondary structure) and *trans*-acting splicing factors that dictate where and in what context differential splicing will occur in transcripts (Wang and Burge, 2008; Barash et al., 2010). The combination of detailed biochemical experiments with more recent genome-wide approaches and computational analyses have revealed diverse mechanisms by which alternative splicing can occur, and have been described in greater detail in several recent excellent reviews (Blencowe, 2006; Chen and Manley, 2009; Licatalosi and Darnell, 2010; Han et al., 2011; McManus and Graveley, 2011; Irimia and Blencowe, 2012).

In this focused review, we examine the role of alternative splicing during neuronal development and in response to neuronal activity. Although a large number of alternative isoforms derived from important neuronal genes have been reported in the literature, special emphasis is given here to recent findings illuminating the role of specific *trans*-acting splicing factors and select target splicing events they regulate in the biogenesis and function of neurons.

## Alternative Splicing in Neuronal Development and Maintenance

**Figure 1:**
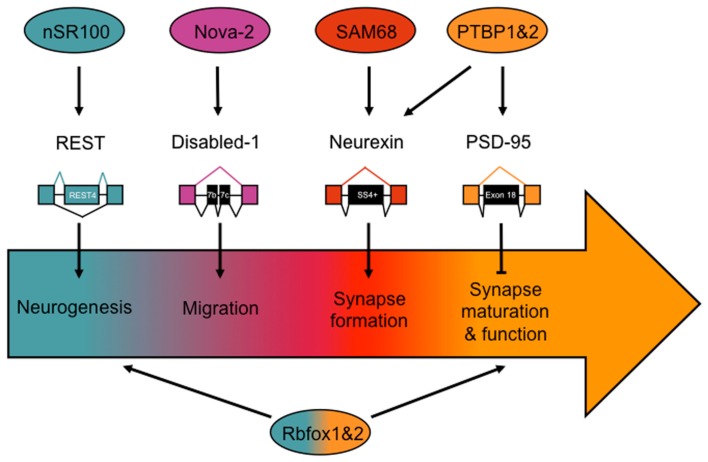
**Notable alternative splicing events important for nervous system development, and the factors implicated in regulating their splicing patterns**. Developmental stages are represented by the arrow, beginning with neurogenesis, and ending with synapse maturation and function. Splicing factors and target transcripts are correspondingly color coded to the developmental stage in which they are known to be important.

Alternative splicing plays an important role in generating diversity and specificity in the developing and mature nervous system. In this section we describe several recent examples demonstrating the role of alternative splicing in neuronal differentiation, migration and pathfinding, and synapse formation and function (Figure 1).

### The RS domain-containing splicing factor nSR100/SRRM4 controls neurogenesis

The neural-specific SR-related protein of 100 kDa (nSR100/SRRM4) was identified in a computational and expression based survey of genes encoding RS domain-containing proteins (Calarco et al., 2009). In a similar manner as other members of the SR and SR-related family of proteins, nSR100 was found to regulate alternative splicing decisions. Microarray profiling experiments in mouse neuroblastoma cells and tissues revealed that depletion of nSR100 results in increased skipping of alternatively spliced exons normally included in the brain, suggesting that it mainly acts to promote the inclusion of alternative exons. A significant fraction of genes containing these regulated exons are known to be important for regulating neuronal differentiation, raising the possibility that specific splice variants modulated by nSR100 could contribute to this process. Consistent with this notion, nSR100 was found to play a critical role in neuronal differentiation and neurite extension *in vitro* as well as nervous system and sensory organ development in zebrafish embryos *in vivo* (Calarco et al., 2009). The specific mechanism by which nSR100 regulates alternative splicing in the nervous system remains to be elucidated. However, nSR100 was shown to be required for proper inclusion of a target neural-specific exon using *in vitro* splicing extracts, indicating that it plays a direct biochemical role in promoting exon inclusion (Calarco et al., 2009). It was also found that the introns flanking alternative exons regulated by nSR100 are enriched in pyrimidine rich motifs (Calarco et al., 2009). The majority of these motifs are likely recognized by the polypyrimidine tract binding protein PTBP1 and its tissue-specific paralog PTBP2 (also called neural- or brain-enriched n/brPTB). Consistent with a link between these regulators in modulating neural-specific alternative splicing, many nSR100-dependent alternative splicing events are also regulated by PTBP1 and PTBP2.

More recently, it was discovered that nSR100 indirectly controls the steady-state abundance of a network of transcripts in neuronal cells distinct from the population of mRNAs that it regulates at the level of splicing. Depletion of nSR100 in mouse neuroblastoma cells led to decreased levels of hundreds of transcripts, and a subset of these changes were shown to be dependent on repressor element 1 silencing transcription factor (REST, also known as NRSF), a transcriptional repressor of genes involved in neurogenesis (Raj et al., 2011). In neuronal cells, REST transcripts include an additional exon that results in the introduction of a stop codon and production of a truncated protein lacking domains required for its repressive activity. Raj et al. (2011) found that nSR100 plays a critical role in promoting the inclusion of this alternative exon, suggesting that the expression of nSR100 in neurons contributes to the reduced activity of REST upon differentiation to the neural lineage. Importantly however, REST was also found to directly repress nSR100 transcription in non-neuronal cells and thus indirectly inhibit neural-specific alternative splicing. This negative feedback loop between two gene regulatory levels was found to be important for developmental outcomes in the nervous system, as inhibiting nSR100 expression in mouse brain disrupted cortical neurogenesis, preventing neuronal precursor cells from committing to a neuronal fate (Raj et al., 2011). These results are in agreement with previous studies showing that loss of REST de-represses neuronal transcripts in non-neuronal tissues, while REST overexpression inhibits the expression of transcripts in neuronal tissues, which in one study was shown to result in axon pathfinding errors in chick embryos (Chen et al., 1998; Paquette et al., 2000).

### The Splicing factor Nova-2 controls neuronal migration

The Nova RNA binding proteins were among the first tissue-specific regulators of alternative splicing to be identified. Initial studies in knockout mice indicated that Nova-1 plays a critical role in the maintenance of brainstem and spinal cord neurons, likely through the regulation of alternative splicing in these cells (Jensen et al., 2000). Subsequent studies utilizing splicing-sensitive microarray profiling in mice lacking Nova proteins identified a network of brain-specific splicing events coordinated by these factors (Ule et al., 2005). Importantly, transcripts with Nova-regulated exons encoded proteins that were significantly enriched in functions associated with the synapse (Ule et al., 2005). Integrating this alternative splicing regulatory network with genome-wide cross-linking and immunoprecipitation (CLIP; Licatalosi et al., 2008; Zhang et al., 2010) studies identifying *in vivo* Nova binding sites in the transcriptome has provided two key advances. First, these datasets have enabled the formulation of “RNA maps” correlating cognate Nova YCAY *cis*-element locations in pre-mRNA transcripts with effects on splicing regulation, leading to mechanistic insights into how the Nova proteins modulate alternative splicing (Ule et al., 2006). Second, these networks identify target isoforms and pathways that likely contribute to aspects of neuronal physiology. In agreement with this latter point, subsequent phenotypic exploration of the role of target isoforms in Nova knockout mice has identified a role for Nova in modulating slow synaptic inhibition and in neuromuscular junction formation (Huang et al., 2005; Ule et al., 2005; Ruggiu et al., 2009).

Recently, Nova-2 was demonstrated to be important for the migration of late-born cortical and Purkinje neurons in mice (Yano et al., 2010). These migration defects were due largely to the aberrant splicing of transcripts from a single gene, the Reelin signaling adaptor Disabled-1. Reelin signaling is an important pathway regulating neuronal migration in the cortex, cerebellum, and hippocampus. Binding of the Reelin ligand to its receptors ApoER2 and Vldlr leads to phosphorylation of Disabled-1, which recruits various adaptor proteins that mediate cytoskeletal rearrangements and appropriate neuronal migration and positioning (Bar et al., 2000; Ayala et al., 2007). Disabled-1 is thus a critical effector protein in the Reelin signaling pathway.

Nova-2 suppresses the inclusion of Disabled-1 exons 7b and 7c (7bc+), which encode an additional 33 amino acid peptide of unknown function, the inclusion of which could produce a protein isoform with dominant negative activity. Expression of Disabled-1 (7bc+) in the E14.5 mouse cortex was sufficient to cause migration defects similar to the Nova-2 knockout, while Disabled-1 (7bc−) substantially rescued the Nova-2 knockout migration defects (Yano et al., 2010). Thus, Nova-2 controls the sensitivity of neurons to the Reelin signaling pathway, presumably by affecting the balance of Disabled-1 (7bc−), which is activated by the Reelin signaling pathway, and Disabled-1 (7bc+), which, through a currently unknown mechanism, impairs the Reelin signaling pathway. The alternative splicing of Disabled-1 is developmentally regulated, with Nova2-dependent suppression of Disabled-1 (7bc+) highest during the critical window of migration for late-born neurons (E14.5–E16.5), suggesting Nova2-mediated alternative splicing of Disabled-1 as a mechanism to control neuronal sensitivity to Reelin signaling throughout development (Yano et al., 2010).

### PTBP1 and PTBP2 regulate synapse formation and maintenance

PTBP1 and PTBP2 display mutually exclusive patterns of expression in the developing brain, with PTBP1 found in glial and non-neuronal cells, and PTBP2 in neurons (Boutz et al., 2007). This non-overlapping pattern of expression is established by an elegant cross-regulatory network where PTBP1 normally suppresses the inclusion of an exon in PTBP2 transcripts, leading to a non-functional isoform degraded by the nonsense-mediated mRNA decay (NMD) pathway (Boutz et al., 2007; Spellman et al., 2007). In neurons however, PTBP1 is silenced by miR-124, a neuron-specific microRNA, leading to the de-repression of PTBP2 (Makeyev et al., 2007). The consequences of modulating the relative levels of PTBP1 and PTBP2 in neuronal cells have been initially revealed through splicing-sensitive microarray profiling of mouse neuroblastoma cells depleted of these factors (Boutz et al., 2007). Analogous to the Nova-regulated alternative splicing network, PTBP1- and PTBP2-dependent alternative splicing events are frequently found in transcripts expressed from genes with known roles in neuronal differentiation and physiology.

A role for PTBP1 and PTBP2 in regulating the expression of PSD-95, an important scaffolding protein essential for synaptic maturation and plasticity of excitatory neurons, has recently been identified. Overexpression of PTBP1 and PTBP2 in cultured hippocampal neurons was shown to repress synaptic activity, dendritic spine formation, and reduce levels of PSD-95 transcripts (Zheng et al., 2012). This reduced mRNA abundance is caused by PTBP1 and PTBP2 binding to a pyrimidine rich *cis*-element upstream of PSD-95 exon 18, leading to increased exon skipping and the production of a transcript containing a premature termination codon that is targeted for degradation by the NMD pathway. Importantly, the increased expression of PSD-95 in developing neurons in the cortex was found to correlate with three distinct phases of PTBP1 and PTBP2 expression. At the neural progenitor stage, when PTBP1 levels are high, PSD-95 expression is at its lowest. In embryonic neurons, the weaker repressor PTBP2 is more highly expressed while PTBP1 expression is lost, leading to intermediate levels of PSD-95. Finally, in post-natal cortical neurons, PTBP2 is no longer expressed, allowing PSD-95 abundance to reach its highest levels (Zheng et al., 2012). These results indicate that the sequential changes in relative expression of PTBP1 and PTBP2 can allow for distinct splicing regulatory programs to be established at different stages in neuronal maturation.

### Alternative splicing of neurexins and neuroligins in synapse formation and maintenance

A number of studies in recent years have demonstrated the importance of alternative splicing of neurexins and their binding partners neuroligins in establishing and/or maintaining synapses (Boucard et al., 2005; Chih et al., 2006; Graf et al., 2006). Neurexins and neuroligins function as adhesion proteins across the synaptic cleft, and increasing evidence suggest that these factors are central organizing proteins at both glutamatergic and GABAergic synapses in the brain (Craig and Kang, 2007). The Neurexin gene loci are highly complex, with the capacity of generating thousands of potential transcript variants pre-synaptically in mammals through the use of alternative promoters and alternative splicing (Boucard et al., 2005; Chih et al., 2006). The post-synaptic neuroligins also undergo alternative splicing, but to a lesser degree. Several key variants from each of these factors have been functionally characterized in cell culture, leading to the proposal of a trans-synaptic adhesive splicing “code” in which particular neurexin isoforms have specific affinity to particular neuroligin isoforms, and the isoforms utilized in neurons affect the functional properties of the synapse (Boucard et al., 2005; Chih et al., 2006; Graf et al., 2006). For instance, the addition of an alternative exon (B+) to neuroligin 1 decreased its ability to recruit GABAergic synaptic components but increased its glutamatergic synaptic recruitment. This change in activity was due to the reduced binding of neuroligin 1 (B+) isoforms to neurexin variants with splice site #4 selected (SS4+; Chih et al., 2006). Neuroligin (B+) bound neurexin (SS4−) strongly but exhibited only weak binding with neurexin (SS4+), while neuroligin (B−) had strong interaction with both neurexin (SS4+) and (SS4−) isoforms. These results point toward a role of neurexin and neuroligin alternative splicing in shaping the strength and class of synapses (Chih et al., 2006).

Several factors involved in the splicing of neurexin transcripts have been identified. The first was PTBP2, which was demonstrated to suppress selection of SS4 in neurexin-2α (Resnick et al., 2008). More recently it has been demonstrated that the KH domain RNA binding protein SAM68 regulates selection of the SS4 in neurexin 1 and neurexin 3, and that it does so in a neuronal activity-dependent fashion (further discussed below; Iijima et al., 2011).

### Rbfox-1/A2BP1 and Rbfox-2/Rbm9 play a role in neuronal development and function

Members of the Rbfox family of RNA binding proteins display enriched or highly specific expression patterns in the neuromuscular system, and regulate alternative splicing decisions through interactions with the highly conserved *cis*-element (U)GCAUG (Underwood et al., 2005; Zhang et al., 2008; Zhou and Lou, 2008; Sun et al., 2012). Focused biochemical studies and several genome-wide analyses have demonstrated that the Rbfox proteins can function as activators or repressors of splicing, depending on the location of (U)GCAUG elements in target pre-mRNA transcripts (Jin et al., 2003; Zhang et al., 2008; Zhou and Lou, 2008; Sun et al., 2012). Together, these studies have begun to shed light on the relevant networks of transcripts modulated by these factors, although the role of the Rbfox proteins in nervous system development and function *in vivo* has remained somewhat unclear.

Two recent studies from the Black laboratory using Rbfox knockout mice have provided further insight toward the functional importance of these proteins in the nervous system (Gehman et al., 2011, 2012). Deletion of *Rbfox1* specifically in the nervous system of transgenic mice did not seem to have any effects on neuronal development or morphology in the brain. However, loss of *Rbfox1* did lead to spontaneous seizures, increased sensitivity to induced seizures, and increased excitability in neurons of the dentate gyrus. Integration of splicing-sensitive microarray profiling and CLIP-Seq datasets identified alternative splicing events differentially regulated in the brains of *Rbfox1^−/−^* mice, several of which were linked to genes known to be associated with epilepsy and others with roles in synaptic function (Gehman et al., 2011).

In contrast to loss of Rbfox1 in the nervous system, deletion of the gene encoding Rbfox2 in the nervous system led to pronounced defects in cerebellar development. *Rbfox2^−/−^* animals have much smaller cerebella than wild-type littermates, defects in Purkinje cell migration and dendritic arborization, and reduction in the migration and number of granule cells (Gehman et al., 2012). Again, splicing-sensitive microarray profiling experiments were performed, revealing alternative splicing events displaying significant changes upon loss of Rbfox2. Genes with affected exons were associated with neuronal development and function, and a subset of Rbfox2-dependent alternative splicing events were also regulated by Rbfox1, suggesting partial redundancy between the two factors. In agreement with these data, double knockout mice displayed far more severe phenotypes than those observed in either single knockout mutant (Gehman et al., 2012). Finally, in an attempt to separate a possible role for both Rbfox proteins in the mature nervous system from their collective role in development, transgenic animals were generated that deleted these two factors specifically in Purkinje cells. Intriguingly, these double knockout mice possess no gross morphological or developmental abnormalities, but display impaired motor skills and significant reductions in spontaneous firing frequency of Purkinje cells, demonstrating that the Rbfox proteins also play an important role in mature neural circuitry in addition to their contribution to development (Gehman et al., 2012).

## Neuronal Activity-Dependent Alternative Splicing Regulation

### Depolarization causes changes in splicing of neuronal transcripts

Calcium signaling has long been recognized to play an important role in various cellular processes such as muscle contraction and gene transcription, and in neurons it is critical for modulating neuronal activity and for learning and memory (West et al., 2001). Increasing evidence suggests that depolarization-induced calcium influx can also regulate alternative splicing in neurons. One of the early studies showing splicing differences in response to neuronal depolarization utilized the cholinergic agonist pilocarpine administered to the brains of rats. Chronic induction of depolarization with pilocarpine caused altered alternative splicing patterns in a number of key neuronal transcripts in the rat hippocampus and cortex, including tra2-beta, clathrin light chain B, NMDAR1, and *c-src* (Daoud et al., 1999).

A number of further studies using chemical treatments to induce or inhibit calcium signaling in neurons have revealed additional calcium-dependent alternative splicing events. For example, Ania-6, an RNA polymerase II-associated cyclin, exhibited increased inclusion of intron 6 upon glutamate stimulation, but decreased inclusion when stimulated by depolarizing concentrations of KCl. Increased intron inclusion leads to altered protein localization such that the longer isoform is found in nuclear speckles and is associated with hyperphosphorylated RNA Pol II (Berke et al., 2001; Sgambato et al., 2003). In a separate study, mature transcripts encoding SNAP25, a membrane-bound component of the SNARE complex essential for synaptic vesicle fusion, were found to include one of two mutually exclusive alternative exons (5a or 5b). Chronic depolarization of PC12 cells or of cerebellar granule cells by exposure to elevated extracellular K^+^ resulted in altered splicing in which the abundance of the 5b isoform is increased (Hepp et al., 2001).

### Modulation of neuronal activity during circadian rhythms

Another potentially interesting physiological process that influences neuronal activity over sufficiently long timescales to involve new gene synthesis and RNA processing lies in the regulation of circadian rhythms. The connection between regulated splicing and circadian rhythms in the nervous system has been supported by a recent study using RNA-Seq in the *Drosophila* brain, which identified numerous splicing events regulated in response to circadian time or period, including splicing of key circadian genes (Hughes et al., 2012). Furthermore, Sanchez et al. (2010) identified the arginine methyl transferase PRMT5, which methylates arginine residues in the spliceosomal Sm proteins, in a screen for novel genes affecting circadian clock regulation in *Arabidopsis*. Mutations in PRMT5 were found to affect both transcription and alternative splicing of many transcripts, including several components of the circadian clock (Sanchez et al., 2010). The authors further demonstrated that a mutation in the *Drosophila* *prmt5* ortholog causes aberrant circadian-dependent behavior as well as altered mRNA splicing patterns. In both organisms, loss of PRMT5 led predominantly to increased intron retention. Taken together, these findings suggest that PRMT5 directly methylates splicing factors, though an alternative model in which PRMT5 leads to epigenetic changes cannot be ruled out (Sanchez et al., 2010). Continued exploration of the mechanisms controlling circadian regulation of alternative splicing will undoubtedly reveal novel insights.

## Mechanisms of Activity-Dependent Alternative Splicing

**Figure 2:**
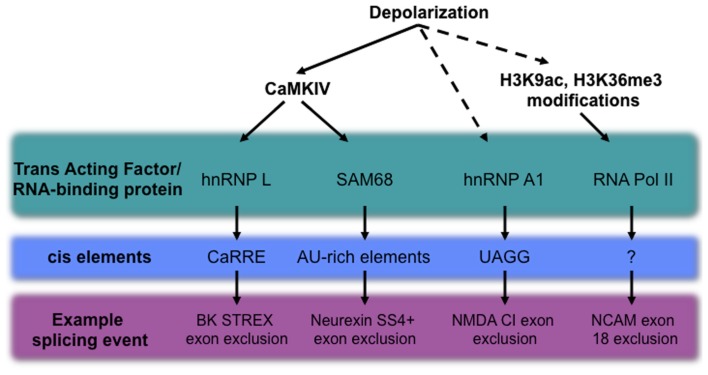
**Mechanisms of depolarization-dependent alternative splicing, including known trans-acting factors, the *cis*-elements with which they interact, and a representative alternative splicing event regulated by each specific pathway**. Established mechanisms are shown as solid arrows, while activities with unknown intermediates are shown with dashed lines.

Although key neuronal transcripts undergoing depolarization-dependent alternative splicing have been discovered, our understanding of the mechanisms controlling this phenomenon is still in its infancy. In the sections that follow, we will highlight current progress in elucidating the role of *cis*-elements, chromatin states, and RNA binding protein modification as regulators of activity-dependent splicing (see Figure 2 for an outline of examples).

### mRNA elements necessary for activity-dependent splicing

Several *cis*-elements regulating alternative splicing changes in response to neuronal depolarization have been identified. The BK (Big Potassium) channel encoded by the *Slo* gene in mammals is important for determining calcium and voltage sensitivity in neurons. The STREX exon of *Slo*, which contributes to enhanced neuronal sensitivity to Calcium when included in transcripts (Saito et al., 1997; Xie and Black, 2001), undergoes increased exon exclusion in response to KCl-mediated depolarization in cultured cells (Xie and Black, 2001). This depolarization-dependent alternative splicing required the Ca^2+^/calmodulin-dependent protein kinase CaMKIV. The *cis*-elements that conferred responsiveness to CAMKIV signaling were identified, and came to be known as CaRREs (CaMKIV-responsive RNA elements; Xie and Black, 2001). Since then, a number of additional depolarization-dependent alternatively spliced transcripts have been shown to contain CaRREs and to be responsive to CaMKIV (Xie et al., 2005; Lee et al., 2007). Candidate RNA binding proteins that act downstream of CaMKIV signaling and bind to the CaRREs had remained elusive until recent years, when the heterogeneous ribonucleoprotein hnRNP L was identified to interact with CaRRE1 at the upstream 3′ splice site of the STREX exon (Yu et al., 2009). Knockdown of hnRNP L led to increased inclusion of the STREX exon prior to depolarization. However, upon treatment with KCl, cells with reduced hnRNP L levels had smaller STREX exon inclusion changes relative to control cells, but still showed some response to depolarization (Yu et al., 2009). These results suggest that other factors in addition to hnRNP L play a role in the observed depolarization-mediated effects. Indeed, a more recent study has now demonstrated that hnRNP L-like (hnRNP LL) acts redundantly with hnRNP L for the complete modulation of the STREX exon in response to depolarization (Liu et al., 2012).

An additional *cis*-element involved in activity-dependent splicing was uncovered in experiments demonstrating that K^+^-induced alternative splicing of the CI cassette exon of the NMDA R1 receptor relied on the presence of two exonic UAGG silencing motifs. These motifs were previously identified as part of a multicomponent regulatory code involving 5′ splice site proximal GGGG elements in coordinating tissue-specific splicing regulation of the CI exon (Han et al., 2005). Introduction of the UAGG silencing motif into a constitutively spliced exon in an unrelated gene led to an increase in exon skipping, and importantly, further increased skipping in response to extracellular K^+^-induced depolarization (An and Grabowski, 2007). Biochemical studies demonstrated that the association of the heterogeneous nuclear ribonucleoprotein hnRNP A1 with these UAGG motifs was increased in response to cellular excitation (An and Grabowski, 2007). Although it is currently unclear how this increased association is induced, these results suggest that a signaling cascade must exist that connects responses to depolarization at the plasma membrane and in the cytoplasm with subsequent effects on activity in the nucleus. Interestingly, hnRNP A1 has been demonstrated to alter its shuttling state between the nucleus and cytoplasm in response to osmotic shock (Allemand et al., 2005). It will be interesting to determine whether depolarization leads to increased shuttling of hnRNP A1 to the nucleus, which would offer a possible explanation for the elevated association with UAGG elements observed.

### Modification of chromatin

Nucleosome positioning and chromatin modification have been recognized as important factors in memory formation and consolidation (Guan et al., 2002; Levenson et al., 2004). More recently, chromatin state and epigenetic marks, including post-translational histone-tail modifications and DNA methylation, have been found to play an important role in alternative splicing regulation (Hnilicova and Stanek, 2011; Luco and Misteli, 2011). Genome-wide analyses have indicated that nucleosomes have increased occupancy in exons compared to flanking intronic regions, and that local enrichment of certain histone modifications can facilitate alternative exon inclusion (see below; Nahkuri et al., 2009; Schwartz et al., 2009; Tilgner et al., 2009; Huff et al., 2010). Conversely, splicing has also been found to be important for the establishment of histone H3 lysine 36 methylation in intron-containing genes, suggesting a bi-directional communication (de Almeida et al., 2011; Kim et al., 2011). Additionally, and not mutually exclusive with the influence of chromatin state, the rate of RNA polymerase II (pol II) elongation during nascent transcript synthesis has also been found to affect alternative splicing (de la Mata et al., 2003). Two models have been proposed to possibly account for how pol II elongation can regulate alternative splicing. First, the recruitment model suggests that splicing factors can directly associate with pol II, likely via the C-terminal domain (CTD) of its largest subunit. These pol II-recruited splicing factors would then be available to recognize cognate *cis*-elements found in nascent pre-mRNA transcripts. Second, the kinetic model posits that chromatin structure influences the local rate of pol II transcription elongation, potentially exposing normally weak splice sites for extended periods of time, thereby allowing them to be more efficiently recognized by the spliceosome (Kornblihtt, 2007; Munoz et al., 2010).

Several recent studies have reinforced the hypothesis that local enrichment of distinct histone marks and DNA methylation status at alternative exons and flanking sequences can modulate pol II elongation rate and alternative splicing. First, Luco et al. (2010) have demonstrated using chromatin-immunoprecipitation assays that H3-K36me3 marks are enriched around a set of alternative splicing events regulated by PTBP1. The histone-tail binding protein MRG15, which specifically recognizes H3-K36me3, was also found to be in a physical complex with PTBP1, thus providing a link between histone modifications and the potential recruitment of splicing factors (Luco et al., 2010). In a separate study, Shukla et al. (2011) discovered that the DNA-binding protein CCCTC-binding factor (CTCF) can mediate local pausing of pol II and inclusion of weak alternative exons. These authors further revealed that the action of CTCF is inhibited by DNA methylation at these regulated exons, suggesting a mechanistic link between elongation rate, methylation, and CTCF binding in modulating alternative splicing. Finally, Close et al. (2012) have identified a novel polymerase-associated complex called DBIRD that was found to promote exon skipping. Depletion of components of the DBIRD complex was found to predominantly increase pol II occupancy surrounding regulated exons, leading to more inclusion. In a search for *cis*-elements associated with these DBIRD-sensitive exons, the authors identified enrichment of (A + T) rich sequences, which have been shown in previous studies to act as pol II elongation pause sites *in vitro* (Close et al., 2012).

An intriguing study has also implicated neuronal depolarization in the control of alternative splicing by affecting RNA Pol II transcription kinetics. Exon 18 of Neural Cell Adhesion Molecule (NCAM) transcripts undergoes developmentally regulated alternative splicing in which the exon-excluded isoform (NCAM140) is abundant in neuronal precursors while the exon-included isoform (NCAM180) is increasingly expressed throughout the process of neuronal differentiation (Pollerberg et al., 1985, 1986; Cunningham et al., 1987). Schor et al. (2009) demonstrated that NCAM exon 18 skipping increased in response to neuronal depolarization with KCl. This depolarization-mediated increase in exon skipping was not dependent on CaMKIV (Schor et al., 2009), but instead involved histone modification changes specifically in the vicinity of exon 18. Depolarization led to an increase in histone H3-K9 acetylation and H3-K36 tri-methylation exclusively in the region between exons 17 and 19, as well as a local increase in chromatin relaxation and accessibility. Furthermore, exon 18 inclusion could be artificially recapitulated by either using a mutant “slow” pol II or by applying the drug trichostatin which inhibits histone deacetylation. These results are consistent with a kinetic coupling model in which depolarization leads to specific local histone modifications in the region of the alternative exon causing local chromatin relaxation, in turn increasing the speed of pol II transit through the exon and facilitating increased exon 18 exclusion (Schor et al., 2009).

### Regulation of RNA binding splicing factors

The activity or levels of RNA binding proteins that regulate alternative splicing can be modified in response to neuronal activity. For instance, phosphorylation of hnRNP L on serine 513 by CaMKIV was found to play a crucial role in the differential regulation of STREX exon inclusion upon treatment of cells with KCl (Liu et al., 2012), providing a molecular link between signaling downstream of the stimulus and subsequent effects on alternative splicing. Two recent studies have implicated the RNA binding protein Rbfox-1/A2BP1 as an important splicing factor mediating activity-dependent alternative splicing (Lee et al., 2009; Amir-Zilberstein et al., 2012). First, transcripts encoding Rbfox-1 itself were identified as a target of depolarization-dependent splicing in mouse cells, where exon 19 was found to be excluded in response to depolarization. Exclusion of exon 19 led to the accumulation of a Rbfox-1 protein isoform targeted to the nucleus, where it re-activated the inclusion of target exons initially displaying more skipping upon depolarization (Lee et al., 2009). Thus, Rbfox-1 lacking exon 19 counteracted the effects of depolarization-dependent exon exclusion, suggesting a novel feedback-based mechanism for adapting to chronic neuronal depolarization. A second study implicated Rbfox-1 as an important downstream target of the hypothalamic transcription factor Orthopedia (Otp) in response to stress (Amir-Zilberstein et al., 2012). Rbfox-1 was demonstrated to be a transcriptional target of Otp, and Rbfox-1 transcript levels were upregulated in response to stress in mice. Rbfox-1 in turn was responsible for an increase in exon 14 inclusion in the pituitary adenylate cyclase-activating peptide (PACAP) receptor PAC1. Inclusion of PAC1 exon 14 led to a decrease in the levels of stress-induced corticotropin-releasing hormone (CRH). These results suggest that Rbfox-1-mediated inclusion of PAC1 exon 14 creates an isoform that helps terminate stress-induced transcription of CRH. In agreement with this model, zebrafish injected with morpholinos inhibiting PAC1 exon 14 inclusion exhibited abnormal “anxiety like” behavior and sustained expression of CRH transcription (Amir-Zilberstein et al., 2012).

Another recent study has provided insight into the mechanism controlling depolarization-dependent splicing of the neurexin SS4+ alternative isoform. As discussed above, alternative splicing of SS4 can modulate the affinity of neurexins for their post-synaptic ligands. Iijima et al. (2011) have now shown that SS4 selection can be suppressed in response to induced depolarization by various methods in cultured mouse neurons. This suppression of exon inclusion is dependent on CaMKIV and results in altered *trans*-synaptic signaling in response to depolarization. The STAR family RNA binding protein SAM68 was shown to be required for depolarization-dependent splicing of SS4, and to bind directly to AU-rich response elements in the neurexin pre-mRNA. Although SAM68 protein levels and localization were not affected by depolarization, a serine residue within a consensus CaMKIV recognition motif was found to be more highly phosphorylated following depolarization. These results suggest a model where neuronal depolarization affects CaMKIV due to increased intracellular calcium, leading to the phosphorylation of SAM68, which then alters neurexin splicing. Interestingly, the neurexin pre-mRNA does not contain recognizable CaRRE sequences, and a number of transcripts containing CaRRE sequences were not affected by loss of SAM68 (Iijima et al., 2011). Thus, it appears that CamKIV-dependent alternative splicing regulation depends on multiple downstream RNA binding proteins binding to distinct *cis*-elements.

## Perspectives and Future Directions

Neuronal depolarization can affect the splicing of many transcripts in the nervous system, but the mechanisms by which it does so still remain largely unknown. A major future challenge will be to identify the signaling cascades in addition to the CAMKIV pathway linking cellular excitation to alternative splicing via changes in the activity of splicing factors, chromatin state, and perhaps additional mechanisms. Moreover, experiments thus far revealing a role of depolarization in nervous system alternative splicing have relied on *in vitro* cell culture models requiring chronic depolarization for many hours to modulate the firing activity of neurons. It will now be important to understand how chronic neuronal depolarization affects alternative splicing regulation *in vivo*, and what consequences the affected isoforms have on neuronal physiology. Many depolarization-responsive alternative splicing events that have been identified are found in transcripts encoding channel proteins, neurotransmitter receptors, and other modulators of synaptic strength (Xie and Black, 2001; Lee et al., 2007; Iijima et al., 2011). Phenomena such as synaptic gain control and homeostasis, where synapses can alter their sensitivity in response to chronic hyper- or hypo-stimulation (Burrone and Murthy, 2003), are thought to occur over the course of hours. This time-frame overlaps well with the temporal dynamics of depolarization-induced alternative splicing changes observed *in vitro*. While it may be a technically challenging feat, genome-wide analyses of splicing changes in organisms maintained under differing stimuli or behavioral paradigms inducing such synaptic gain control or homeostatic maintenance would provide further insight into the mechanisms and relevance of neuronal activity in regulating alternative splicing *in vivo*.

The fact that the activity of master regulators of gene expression such as transcription factors can be modified by alternative splicing has blurred the lines of how differentiation programs in cell lineages are established (Gabut et al., 2011; Raj et al., 2011). In a broader sense, these results raise an interesting implication, namely, that regulation of alternative splicing events by RNA binding proteins can play a causal rather than simply consequential role in developmental transitions and activity states of neurons. Future experiments establishing the contribution of splicing and other RNA binding regulators to the identity and fate of neuronal lineages represents an important goal in basic research but also in biomedical applications such as regenerative medicine. While a handful of neuronal-specific splicing regulators have been discovered, it is unlikely that the current repertoire of known factors is sufficient to account for the remarkable degree of splicing complexity observed in the nervous system. Hundreds of RNA binding proteins have been identified in metazoan genomes, and many of them remain uncharacterized. As such, identification and characterization of novel regulators of splicing will be important. Large scale RNAi and/or cDNA overexpression studies in cell culture models will be useful for identifying factors whose inhibition or overexpression affect splicing in the nervous system. Importantly, invertebrate model organisms provide a valuable platform for performing forward and reverse genetic screens to identify previously uncharacterized factors affecting splicing. In addition to identifying RNA binding proteins, such unbiased screens may also uncover novel classes of genes not previously known to regulate alternative splicing, such as non-coding RNAs and chromatin regulators (Luco et al., 2010; Tripathi et al., 2010). Furthermore, it will be important to understand splicing regulation in the nervous system in a more spatially resolved manner, for example, by identifying brain sub-region and neuronal-subtype specific alternative splicing events, the factors that control these events, and the effects they have on specification and function of individual classes of neurons.

Although progress has been made toward understanding splicing changes in the development of the nervous system, much less is known about the interplay between alternative splicing regulation and aging in the brain. It has been known for some time that aberrant splicing of the LMNA gene leads to an accelerated aging phenotype found in individuals with Hutchinson-Gilford Progeria Syndrome (HGPS; Todorova et al., 2003). Recent studies using patient cell lines and HGPS mouse models have identified candidate regulators involved in the cryptic splicing of LMNA (Lopez-Mejia et al., 2011). It is tempting to speculate that analysis of the aging brain will also implicate splicing factors in both aging-related splicing changes and the gradual deterioration of the nervous system. Indeed, a recent study has identified dynamic alterations in splicing during normal brain aging consistent with an increase in PTB-dependent splicing, as well as splicing changes in diseased brain consistent with decreased NOVA-dependent splicing (Tollervey et al., 2011). In the future, it will be important to more directly understand the mechanisms and the consequences of splicing in the aging brain.

Finally, mutations in several RNA binding proteins expressed in the nervous system have been associated with neurodevelopmental disorders (Grabowski and Black, 2001; Wang and Cooper, 2007; Morikawa and Manabe, 2010). Advances in genome-wide approaches to globally monitor transcripts bound by these proteins and their effects on aspects of mRNA metabolism, including alternative splicing, are beginning to shed light on underlying mechanisms of action and provide a more detailed understanding of disease etiology. Deeper investigation of the transcript networks regulated by these RNA binding proteins will hopefully provide promising new insight into the development of treatments for some of these disorders.

As demonstrated by some of the examples described above, alternative splicing has the potential to generate multiple protein isoforms, but can also modulate other properties of mRNA transcripts, including their stability. Two recent studies have suggested that tissue-specific alternative exons can frequently encode structurally disordered regions in proteins and have the potential to influence post-translational modification and protein–protein interaction interfaces (Buljan et al., 2012; Ellis et al., 2012). These observations collectively indicate that it remains an important goal to develop additional techniques and approaches that will facilitate the characterization of the functional consequences of alternative splicing events in biological pathways.

We are embarking on an exciting time where techniques for large scale analysis of nervous system transcriptome dynamics in distinct cellular subtypes, multiple developmental states, and in response to environmental cues, are constantly improving. These approaches are already revealing a previously unappreciated role for post-transcriptional gene regulatory mechanisms in almost all aspects of nervous system physiology. Continued research taking advantage of these techniques, coupled with emerging computational approaches and more traditional biochemical and molecular genetic assays, will produce a more comprehensive understanding of alternative splicing regulation. These integrative analyses should also shed further light on the interplay between alternative splicing and other layers of gene regulation in generating the constellation of neuronal subtypes and their diverse functional properties.

## Conflict of Interest Statement

The authors declare that the research was conducted in the absence of any commercial or financial relationships that could be construed as a potential conflict of interest.
